# Metformin inhibition of neuroblastoma cell proliferation is differently modulated by cell differentiation induced by retinoic acid or overexpression of NDM29 non-coding RNA

**DOI:** 10.1186/1475-2867-14-59

**Published:** 2014-07-02

**Authors:** Delfina Costa, Arianna Gigoni, Roberto Würth, Ranieri Cancedda, Tullio Florio, Aldo Pagano

**Affiliations:** 1 Department of Experimental Medicine (DIMES), University of Genova, Genova, Italy; 2 Internal Medicine (DIMI), University of Genova, Genova, Italy; 3 IRCCS-AOU San Martino-IST, Genova, Italy; 4 Center of Excellence for Biomedical Research (CEBR), University of Genova, Genova, Italy

**Keywords:** Metformin, Neuroblastoma, NDM29 ncRNA, Differentiation, Anti-cancer therapy

## Abstract

**Background:**

Metformin is a widely used oral hypoglycemizing agent recently proposed as potential anti-cancer drug. In this study we report the antiproliferative effect of metformin treatment in a high risk neuroblastoma cell model, focusing on possible effects associated to different levels of differentiation and/or tumor initiating potential.

**Methods:**

Antiproliferative and cytotoxic effects of metformin were tested in human SKNBE2 and SH-SY5Y neuroblastoma cell lines and in SKNBE2 cells in which differentiation is induced by retinoic acid treatment or stable overexpression of NDM29 non-coding RNA, both conditions characterized by a neuron-like differentiated phenotype.

**Results:**

We found that metformin significantly inhibits the proliferation of NB cells, an effect that correlates with the inhibition of Akt, while AMPK activity resulted unchanged. Notably, metformin effects were modulated in a different ways by differentiating stimuli, being abolished after retinoic acid treatment but potentiated by overexpression of NDM29.

**Conclusion:**

These data suggest the efficacy of metformin as neuroblastoma anticancer agent, and support the requirement of further studies on the possible role of the differentiation status on the antiproliferative effects of this drug.

## Introduction

Metformin (N,N-dimethylbiguanide) is an oral biguanide in clinical use since the 1950s for its hypoglycemic activity. Currently, it is the most widely used anti-type 2 diabetes drug, with nearly 120 million prescriptions worldwide filled every year [1]. Metformin decreases hyperglycemia primarily by activating 5’-adenosine monophosphate-activated protein kinase (AMPK) to suppress glucose production in the liver, increase glucose utilization, and reduce hyperinsulinemia [2].

Recent population studies suggested that metformin decreases the incidence of several cancers and cancer-mortality in diabetic patients [3-6], and improves the response to chemotherapy in diabetic breast cancer patients [7]. Additionally, this therapeutic potential has been confirmed *in vitro* since metformin inhibits in breast, colon, lung, prostate, and pancreas cancer cell proliferation [8-11]. These studies highlight a direct antitumoral activity of metformin, besides the possible indirect effects mediated by the improvement of the metabolic parameters and, in particular, of the hyperinsulinemia. More recently, prospective studies also demonstrated that preoperative metformin treatment of non-diabetic patients with breast (two weeks) or colorectal aberrant cryptic foci (one month) provided a reduction of the number of proliferative cells [12,13].

Interestingly, it was shown that the antitumor effect exerted by metformin in breast cancer, glioblastoma, and hepatocellular carcinoma cells is mainly mediated by a directed and selective antiproliferative activity against the cancer stem/tumor initiating cell (TIC) fraction [14-17]. According to the cancer stem cell theory this cell subpopulation represents the main pharmacological target to obtain efficacious therapeutic responses in tumors [18-20].

In this work we address, for the first time, the possible anticancer effect of metformin in a high risk neuroblastoma (NB) cell model, including cancer cell lines displaying different levels of differentiation and stemness/tumor initiating potential.

In particular, we document a significant inhibition of NB cells proliferation and viability exerted by metformin. Interestingly, overexpression of NDM29, a NB differentiating non-coding (nc)-RNA, transcribed by RNA polymerase III, and able to reduce cell tumorigenicity [21-23], leads to an increased cell sensitivity towards metformin, while all trans-retinoic acid (ATRA)-induced differentiation reduced metformin NB cell susceptibility.

These findings provide the basis for further, deeper investigations on the possible usefulness of metformin as adjuvant/neo-adjuvant treatment for NB, and its specific role in the stemness/differentiation balance of tumor cells.

## Materials and methods

### Cell Cultures and metformin treatment

Cell lines: SH-SY5Y, grown in DMEM (Sigma–Aldrich), supplemented with 10% FBS (GIBCO), L-glutamine (2 mM; EuroClone), and penicillin–streptomycin (100 U/ml/ 100 μg/ml; EuroClone); SKNBE2, grown in RPMI (Sigma–Aldrich), supplemented with 10% FBS (GIBCO), L-glutamine (2 mM; EuroClone), and penicillin–streptomycin (100 U/ml/ 100 μg/ml; Euro Clone).

SKNBE2 cells were transfected using polyethylenimine (PEI; Sigma P3143) with pEGFP-N1 as control (hereafter referred to as pMock) or pEGFP-N1-NDM29 (hereafter referred to as NDM29). G418 (geneticin; Invitrogen) was used in culture medium as mean of selection up to 1000 μg/ml, until resistant clones were identified. After selection, the clones were preserved in 200 μg/ml G418 in standard culture conditions. Treatment with metformin (20 mM) was performed when cell culture reached 80% of confluence. ATRA treatment was performed in SKNBE2 and SHSY5Y neuroblastoma cells grown in RPMI or DMEM medium with 10% FBS. Cells were grown for 2 days to reach the log phase of growth. When cell cultures reached 80% of confluence the medium was replaced with RPMI or DMEM medium containing 10% FBS and ATRA (1 or 10 μM) or DMSO (0.01% or 0.1%) in control cultures. Cells were then grown for 10 days before the experiments were performed.

### Cell proliferation and cytotoxicity assays

A) Real time cell proliferation and cytotoxicity was assessed by xCELLigence RTCA DP System (Roche, Germany), as reported [24]. This system monitors cellular events in real time by measuring electrical impedance across interdigitated gold micro-electrodes integrated on the bottom of tissue culture plates. The impedance measurement provides quantitative information about the biological status of the cells, including cell number, viability, and morphology. Cell-sensor impedance is expressed as an arbitrary unit called Cell Index [25]. In order to calculate CI, cells were seeded into 100 μL of standard medium in 96X microtiter plates (E-Plate-Roche, Germany). Background impedance was determined using 50 μl of standard medium. After 24 hrs, 20 mM metformin was added to the wells and cell proliferation was monitored for 72 hrs or more. Cell adhesion, spreading and proliferation were monitored every 30 min using the xCELLigence system to produce time-dependent cell response dynamic curves. All experimental results were obtained using RTCA Software 1.2 of the xCELLigence system.

B) Cell counting studies: cell from the different lines were plated in 6-well plates, incubated in standard medium for approximately 24 hrs before being treated with 20 mM metformin. Cells were counted with a hemocytometer after 24 and 48 hrs of metformin treatment and evaluated by Trypan blue exclusion test in which live cells are able to exclude the dye from the cytosol.

C) [^3^H]-thymidine incorporation assay: different cell lines were plated in 24-well plates, incubated in standard medium for 24 hrs, then treated with 20 mM metformin. After 24 and 48 hrs of metformin treatment, cells were pulsed with [^3^H]-thymidine (0.3 μCi/500 μl/well) (GE Healthcare, New York, NY) for the last 14 hrs. Averaged proliferation rate was then calculated by the thymidine uptake assay.

D) ATPlite 1step Luminescence assay: this system measures cellular ATP levels, as a marker of cell viability (Perkin Elmer, Monza, Italy). ATPlite 1step assay system is based on the production of light caused by the reaction of ATP with added luciferase and D-luciferin. The emitted light is proportional to ATP concentration. Cell suspension (100 μl) was seeded in 96-well culture plate white (Thermo Scientific). After 24 hrs, 100 μl culture medium containing metformin (20 mM) was added to the cells. Each group had 5 repeats. The plates for the ATP-Lite assay were incubated for 24 or 48 hrs at 37°C. Then 100 μl of culture medium was removed from each well using a manual multichannel pipet, followed by the addition to each well of 100 μl cell substrate solution (ATPlite kit content). The plate was shaken for 2 min followed by dark adapted for 10 min and luminescence was measured using a luminometer (TECAN Genios Pro reader).

E) SYTOX Blue dead cell stain: this is a high-affinity nucleic acid stain that easily penetrates dead cells with compromised plasma membranes but not in the healty ones. Cells were plated in 6 well-plates, incubated in standard medium for 24 hrs, and treated with 20 mM metformin for 24 or 48 hrs at 37°C. After brief incubation with SYTOX Blue stain, nucleic acids of dead cells fluoresce bright blue when excited with 405 nm violet laser light. Samples were analyzed using a Cyan ADP cytofluorimeter (Beckman-Coulter, Brea CA, USA). For each sample, 20,000 events were acquired. The data were analyzed using Summit 4.3.1 software (Beckman-Coulter, USA).

### Western blotting

Cells were plated onto 60-mm dishes for 24 hrs before being treated. Cells were lysed in buffer containing 1% Nonidet P-40, 20 mM Tris–HCl, pH 8, 137 mM NaCl, 10% glycerol, 2 mM EDTA, 1 mM phenylmethylsulfonyl fluoride, 1 mM sodium orthovanadate, 10 mM NaF (all from Sigma), and the “Complete” protease inhibitor mixture (Roche Applied Science) for 10 min at 4°C [26]. Nuclei were removed by centrifugation (13,200 rpm at 4°C, for 2 min), and total protein content was measured using the Bradford assay (Bio-Rad). Proteins (20 μg) were resuspended in 2X reducing sample buffer (2% SDS, 62.5 mM Tris, pH 6.8, 0.01% bromphenol blue, and 1.43 mM β-mercaptoethanol, 0.1% glycerol), electrophoresed on 10% SDS polyacrylamide gels, transferred on polyvinylidene difluoride membrane (Bio-Rad), and probed with specific antibodies directed against total and phospho-Akt (Ser-473), total and phospho-AMPk, total and phospho-mTOR, and procaspase-3 and cleaved caspase-3, all purchased from Cell Signaling Technology (Beverly, MA); α-tubulin (Sigma) was used as protein loading standard. Immunocomplex detection was performed using ECL system (Bio-Rad), as reported [27].

### Real time quantitative RT-PCR analysis

Total RNAs from samples were extracted using TRIzol reagent (Invitrogen) according to the manufacturer’s protocol, DNAseI-digested and subjected to reverse transcription by Transcriptor High Fidelity cDNA Synthesis Kit (Roche 05081955001) following manufacturer’s instructions. The total RNA from samples was measured by real-time quantitative RT-PCR using PE ABI PRISM@ 7700 Sequence Detection System (Perkin Elmer Corp./Applied Biosystems, Foster City, CA) and SybrGreen method. The sequences of forward and reverse primers were: NDM29, 5’-GGCAGGCGGGTTCGTT-3’ and 5’-CCA CGCCTGGCTAAGTTTTG-3’; neurofilament-68 (NF-68), 5’-CAAGGACGAGGTGTCCG AG-3’ and 5’-CCCGGCATGCTTCGA; Integrin beta-1 (CD29), 5’-AACGAGGTCATGGTT CATGTTG-3’ and 5’-ACCACACCAGCTACAATTGGAA-3’. For endogenous control, the expression of glyceraldehyde 3 phosphate dehydrogenase (GAPDH) gene was examined. The sequences for human GAPDH rRNA forward and reverse primers were 5’-GAAGGTGAAG GTCGGAGTC-3’ and 5’-GAAGATGGTGATGGGATTTC-3’. Relative transcript levels were determined from the relative standard curve constructed from stock cDNA dilutions, and divided by the target quantity of the calibrator following manufacturer's instructions.

### Statistical analysis

Experiments were performed at least in triplicate. Data are reported as mean values ± standard error (SE). Statistical significance of observed differences among different experimental groups was examined using the unpaired Student's t-test, as reported [28]. Statistical analysis were performed before normalization (metformin *vs* control).

## Results

### Metformin inhibits neuroblastoma cell growth

**Figure 1:**
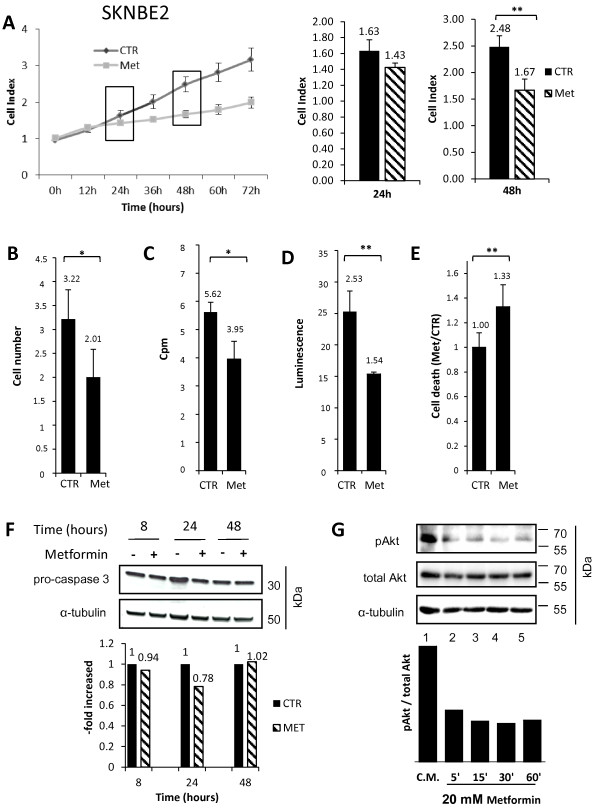
**Metformin reduces cell proliferation and viability of Neuroblastoma SKNBE2 cells. A)** Cell Index curves reporting SKNBE2 neuroblastoma cell proliferation, untreated (CTR) or treated with metformin (Met), as resulting by Xelligence RTCA DP analysis. Histograms highlight Cell Index values after 24 and 48 hrs. **B)** Cell counting assay of SKNBE2 cells untreated (CTR) or treated with metformin (Met). Y-axis is referred to cell number x 10^6^. **C)** [^3^H]-thymidine incorporation assay of SKNBE2 cells, untreated (CTR) or treated with metformin (Met). Y-axis is referred to cpm x 10^3^. **D)** Cell viability analysis of SKNBE2 cells untreated (CTR) or treated with metformin (Met) as determined by ATPlite assay. Y-axis is referred to luminescence x 10^5^. **E)** Cell death analysis by FACS analysis for sytox blue staining. The percentage of cell death is reported as Met/CTR ratio. **F)** Western blot analysis of caspase-3 cleavage. Equal loading of proteins was ensured by normalization for pro-caspase 3 and α-tubulin expression. Cleaved Caspase 3 was not detected. **G)** Western blot analysis of Akt phosphorylation/activation. 1 = Complete Medium (C.M., RPMI 10%), 2 = C.M. + 20 mM Met for 5 min, 3 = C.M. + 20 mM Met for 15 min, 4 = C.M. + 20 mM Met for 30 min, 5 = C.M. + 20 mM Met for 60 min. Equal loading of proteins was ensured by normalization for total Akt and α-tubulin expression. Densitometric analysis of phospho-Akt/total Akt ratio is also reported. (*p < 0.05; **p < 0.01).

In order to investigate the effect of metformin on NB, SKNBE2 cells were treated for 12–72 hrs with metformin (20 mM) and proliferation rate analyzed using multiple experimental approaches. First, we measured cell proliferation using the xCELLigence RTCA DP system. SKNBE2 growth curves showed a time-dependent decrease in the proliferation rate of metformin-treated cells, resulting in a statistically significant difference after 48 hrs (as evidenced by the calculated Cell Index, see Methods), and lasting up to the end of the experimental observation (72 hrs) (Figure 1A).

In order to evaluate whether the reduction in Cell Index was directly associated to a decrease of cell proliferation, control and metformin-treated cells were counted using an hemocytometer. Results confirmed a significant reduction of the cell number 48 hrs after metformin administration (Figure 1B). In addition, a statistically significant reduction of the mitotic rate of metformin-treated SKNBE2 cells was confirmed by a statistically significant decrease in DNA synthesis 24 hrs after the treatment, measured by [^3^H]-thymidine incorporation (Figure 1C). Next, metformin effects on cell viability were tested using the ATPlite assay, which, measuring intracellular ATP content in metabolically active cells, is an indirect index of cell viability. In agreement with the previously described experiments, we found that, after 48 hrs of treatment, metformin leads to a statistically significant decrease in ATP content (Figure 1D).To strengthen these results, and verify whether metformin effects were merely cytostatic or cytotoxicity was also induced, we quantified, by FACS analysis, the percentage of dead cells by SYTOX blue incorporation assay, in which the intracellular accumulation of the dye is an index of dead cells. Again, results showed that metformin increased the number of cell death after 48 hrs of treatment (Figure 1E).In order to assess the mechanism of cell death induced by metformin we tested by Western blot the possible activation of the apoptotic machinery measuring the expression of the cleaved/activated caspase-3. However, 8, 24 and 48 hours of treatment with metformin 20 mM did not increase the cleaved caspase-3 intracellular content and unaltered caspase-3 pro-enzyme expression was detected (Figure 1F). In addition, in treated cells, we observed neither the up-regulation of the pro-apoptotic protein Bax, nor the presence of nuclear piknosis, both common features of apoptotic cells (data not shown). Altogether these results exclude apoptosis from the major causes of cell death induced by metformin.

To investigate at molecular level the intracellular pathways altered by metformin to affect SKNBE2 cells proliferation and survival, we measured its effects on Akt phosphorylation/activation. Akt inhibition, known to correlate with impaired cell survival, was recently shown to represent a molecular correlate of metformin direct antitumoral effects [15]. In Western blot experiments, we report that metformin (20 mM) induced a long-lasting (5–60 min) decline in Akt phosphorylation/activation (Figure 1G), suggesting that metformin effects in SKNBE2 cells were mediated by the inhibition of Akt activity. Conversely, AMPK/mTOR pathways was not affected (see below).

**Figure 2:**
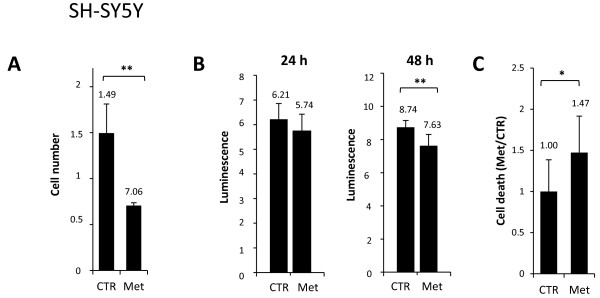
**Metformin antiproliferative effects towards neuroblastoma cell lines with different malignant potential. A)** Cell counting assay in SH-SY5Y cells, untreated (CTR) or treated with metformin (Met). Y-axis is referred to cell number × 10^6^. **B)** Cell viability analysis of SH-SY5Y cells untreated (CTR) or treated with metformin (Met), as determined by ATPlite assay. Y-axis is referred to luminescence × 10^5^. **C)** Cell death quantification by FACS analysis for SYTOX blue staining in untreated (CTR) or metformin (Met)-treated cells. The percentage of cell death is reported as Met/CTR ratio. (*p < 0.05; **p < 0.01).

Since malignant potential of NB nodules is often dependent on N-myc amplification/overexpression (as occurs in SKNBE2 cells), we investigated whether the antitumoral effects of metformin are somehow modulated by the activity of this oncogene. To this purpose we tested metformin antiproliferative activity in the SH-SY5Y NB cell line in which N-myc is not amplified [29]. Metformin (20 mM) treatment induced a statistically significant reduction of proliferation rate after 48 hrs, also in SH-SY5Y cells (Figure 2A). In addition, metformin effect on cell viability was assessed by ATPlite assay, performed after 24 and 48 hrs of treatment. A reduction of cell viability was detected although to a lesser extent respect to SKNBE2 cells, reaching statistical significance after 48 hrs of treatment (Figure 2B). Interestingly, after 48 hrs of treatment, SYTOX blue assay evidenced that metformin-induced cell death in SH-SY5Y cells, although reaching statistical significance, was less pronounced as compared to what observed in SKNBE2 cells (Figure 2C).

Altogether these results suggest that metformin is an effective antiproliferative agent towards NB cell lines with different malignant potential.

### Effects of metformin on differentiated neuroblastoma cell growth

NB is composed by extremely heterogeneous cell populations, including cells at different maturation stages. Thus, we investigated the efficacy of metformin as antiproliferative agent in NB cells at different level of differentiation and malignancy.

**Figure 3:**
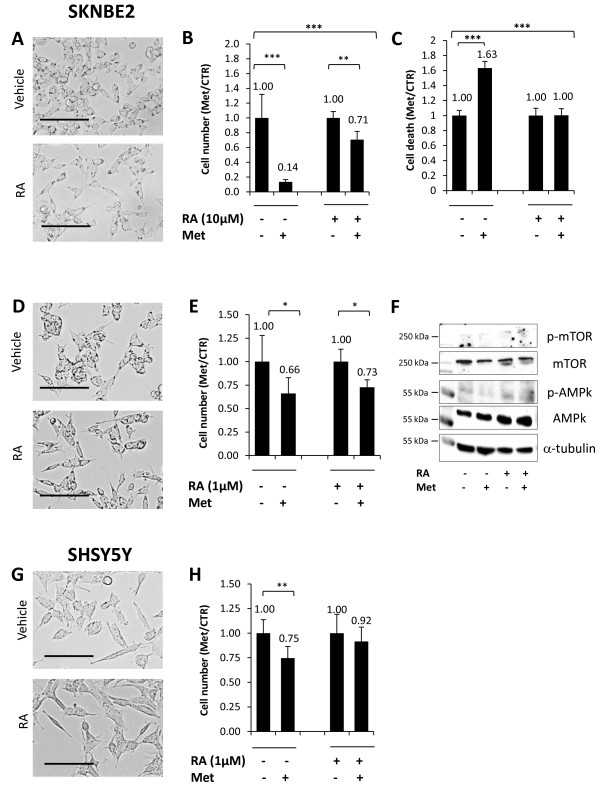
**Retinoic acid-dependent differentiation of SKNBE2 cells decreases metformin antiproliferative effects. A)** Morphological analysis of SKNBE2 cell lines treated with retinoic acid (RA) 10 μM or vehicle (scale bar = 100 μm). **B)** Cell counting assay in SKNBE2 differentiated with 10 μM retinoic acid (RA) and the respective control, in the presence or absence of metformin (Met). The number of cells, are reported as Met/CTR ratio. **C)** Cell death analysis by FACS analysis for SYTOX blue staining. The percentage of cell death is reported as Met/CTR ratio. **D)** Morphological analysis of SKNBE2 cells treated with 1 μM retinoic acid (RA) or vehicle (scale bar = 200 μm). **E)** Cell counting assay of SKNBE2 cells treated with 1 μM retinoic acid (RA) and the respective control, in the presence or absence of metformin (Met). The number of cells, are reported as Met/CTR ratio. **F)** Western blot analysis of AMPk and mTOR phosphorylation/activation induced by metformin (Met) before and after the induction of RA-mediated differentiation of SKNBE2 cells. Equal loading of proteins was ensured by α-tubulin expression. **G)** Morphological analysis of SHSY5Y cells treated with retinoic acid (RA) 1 μM or vehicle (scale bar = 100 μm). **H)** Cell counting assay in SHSY5Y cells, treated with retinoic acid (RA) 1 μM and the respective control, in the presence or absence of metformin (Met). The number of cells, are reported as Met/CTR ratio. Images were taken by EVOS fl, AMG. (*p < 0.05; **p < 0.01; ***p < 0.001).

To this aim we first assessed the effects of the treatment with metformin using SKNBE2 cells differentiated after ATRA treatment (10 μM, for 10 days) that leads to a neuron-like commitment (Figure 3A). Interestingly, in cell count experiments, we found that the remarkable reduction of cell proliferation rate induced by metformin in non-differentiated control cells, was greatly reduced in differentiated SKNBE cells exposed to metformin, resulting in a highly significant statistical difference comparing the two experimental groups (Figure 3B). SYTOX blue assay corroborated this evidence showing, after metformin treatment, a significant increase of death rate in non-differentiated cells, which was almost abolished in SKNBE2 cells differentiated with ATRA (Figure 3C). In order to discriminate between the antiproliferative effect that ATRA exert *per se* and those induced by metformin we treated SKNBE2 cells with a lower dose of ATRA (1 μM, for 10 days). In this condition the treatment induced slight morphological changes accompanied by a weak reduction of metformin susceptibility as showed by cell counting experiments (Figure 3D,E). Therefore, ATRA affects metformin susceptibility in SKNBE cells by inducing NB cells differentiation in a dose-dependent manner.

Since in differentiated hepatic cells metformin hypoglycemic effects are mainly mediated by the modulation of the AMPK/mTOR pathway, we examined whether this may occur also in differentiated NB cells. By western blot analysis we compared the effects of metformin on AMPK and mTOR phosphorylation before and after the induction of ATRA-mediated differentiation in SKNBE2 cells. As reported in Figure 3F, no differences on the activity (phosphorylation) of AMPk or mTOR has been found in both differentiated and undifferentiated cells. Altogether, these results show that the susceptibility to the effects of metformin is directly correlated to NB differentiation level, as reported in glioblastoma cells [15] and confirmed that AMPK seems not involved in the antiproliferative effects of the drug.Next we investigated the occurrence of this effect in NB cells highly responsive to ATRA-induced differentiation. We found that, although less sensitive to metformin than SKNBE2 cells, SHSY5Y cells were highly influenced by ATRA treatment (1 μM, for 10 days) that completely abolished the ability of metformin to reduce cell proliferation (Figure 3G,H). Higher ATRA concentrations (10 μM, for 10 days) did not lead to additional changes in metformin susceptibility (data not shown).

To delve deeper into the mechanisms by which differentiation may affect metformin antiproliferative effects in NB cells, we took advantage of a novel *in vitro* model of NB differentiation, we recently developed, that was based on the expression level of a nc-RNA, named NDM29, in SKNBE2 cells [21,22,30]. In this system the level of differentiation of NB cells, as well as the reduction of the stemness/tumor initiating potential, is directly related to the level of synthesis of NDM29 [21,22,30]. Here, we compared the effects of metformin in mock transfected cells, that express low levels of endogenous NDM29 RNA, with established cell lines in which different levels of NDM29 are overexpressed. The overexpression of ncRNA NDM29 represent a novel model of NB cell differentiation, resulting in the acquisition of neuronal electrophysiological properties as previously reported [22].

**Figure 4:**
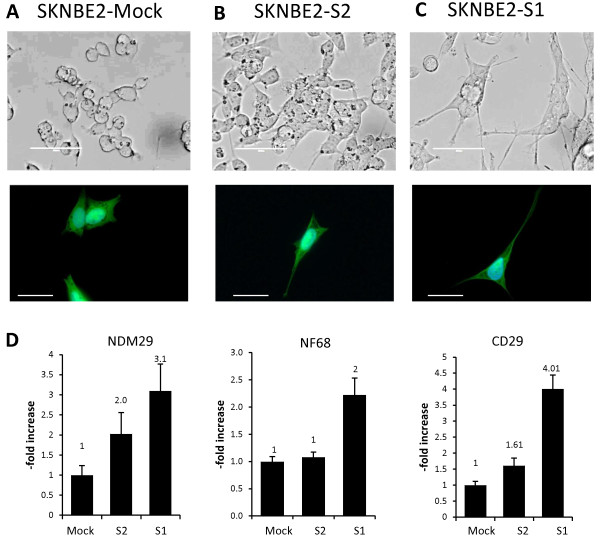
**NDM29 expression level correlates with the differentiation of NB cells toward a neuron-like phenotype. A)** SKNBE2-Mock cells expressing the basal level of NDM29 ncRNA; **B,C)** SKNBE2-S2 and SKNBE2-S1 cells expressing increased level of NDM29 [21]; (scale bar = 50 μm). Upper panels: transmitted light microscopy (EVOS fl, AMG). Lower panels: fluorescence microscopy (Zeiss Axiovert 200 M inverted microscope, Jena Germany). Blue = DAPI, green = GFP (scale bar = 25 μm). **D)** Real time RT-PCR analysis of NDM29, NF-68, (differentiation markers) and CD29 (adhesion marker) expression.

**Figure 5:**
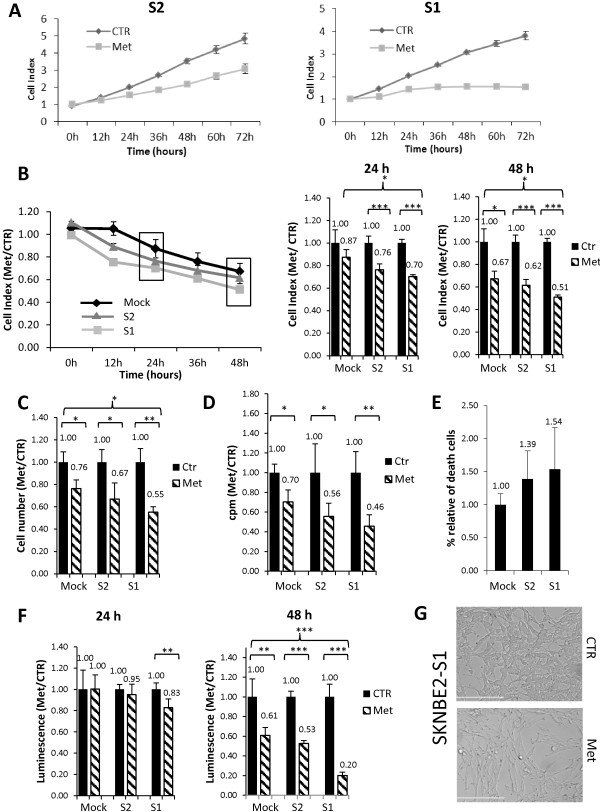
**Higher efficacy of metformin antiproliferative effects in NDM29 differentiated neuroblastoma cell populations. A)** Cell Index curves of the SKNBE2-S2 and SKNBE-S1 NB cell proliferation untreated (CTR) or treated with metformin (Met), as resulting by Xcelligence RTCA DP analysis. **B)** Cell Index analysis of SKNBE2-Mock, SKNBE2-S2 and SKNBE2-S1 NB cells untreated (CTR) or treated with metformin (Met), as resulting by Xcelligence RTCA DP analysis. Data were reported as Met/CTR ratio for each cell line. Histogram on the right panel report statistical analysis of 24 and 48 hrs cell index measures. **C)** Cell counting assay and **D)** [^3^H]-thymidine incorporation assay of SKNBE2-Mock, SKNBE2-S2 and SKNBE2-S1 cells untreated (CTR) or treated with metformin (Met). Cell number **(C)** and cpm **(D)** values are reported as Met/CTR ratio for each cell type. **E)** FACS analysis of cell dead with SYTOX blue stain of different cell lines (SKNBE2-Mock, S2, S1). The percentage of cell death is reported as Met/CTR ratio, for each cell type. **F)** Cell viability determination by ATPlite assay in different cell lines (SKNBE2-Mock, S2 and S1) untreated (CTR) or treated with metformin (Met). Luminescence values, are reported as Met/CTR ratio for each cell type. **G)** Morphology of S1 cells (scale bar = 200 μm) by EVOS fl, AMG. (*p < 0.05; **p < 0.01; ***p < 0.001).

In particular, we used the following permanently transfected cell lines: SKNBE2 cells that express NDM29 at endogenous level (transfected with the empty vector, pMock), NDM29 overexpressing cells, showing increased NDM29 RNA expression 2- (S2) and 3.1-fold (S1) over pMock cells. As depicted in Figure 4, NDM29 stably transfected cells, co-expressing GFP, show a strong enrichment in neuritic processes, acquiring a frank neuronal morphology, associated to the expression of neuronal (neurofilament NF-68) and adhesion (CD29) markers. Importantly, the acquisition of all the differentiation features were proportional to the expression level of NDM29 (Figure 4A-D). Cells from all these lines were treated for 48 hrs with metformin (20 mM) and tested for cell proliferation rate. Surprisingly, by Xcelligence detection system, we observed that metformin was able to affect cell proliferation also in the differentiated S2 and S1 cell lines (Figure 5A). Interestingly, we found that metformin activity was directly correlated to NDM29 expression, which, in turn, determines the differentiation level of the cells. Indeed, higher metformin-dependent inhibition of cell proliferation was observed in well-differentiated S1 cells than in mock-transfected cells (Figure 5B). In order to better associate these effects to cell proliferation rather than an artifactual parameter detected by this system, such as cell area, these data were confirmed in cell counting and [^3^H]-thymidine incorporation experiments showing that metformin inhibition of DNA synthesis showed a higher statistical significance in NDM29-overexpressing cells as compared to mock cells (Figure 5C,D). In agreement with these results, FACS analysis for SYTOX blue staining, after 24 hours of metformin treatment, showed increased SKNBE2 death rate directly correlated with NDM29 expression (Figure 5E), and ATPlite assay showed decreased cell viability, proportional to the levels of NDM29 expression (Figure 5F). Interestingly, microscopic examination revealed a dramatic alteration in the morphology of metformin-treated S1 cells that acquired large, elongated shape recapitulating a behavior previously observed in glioma cell lines (Figure 5G) [31,32].

These results highlight an unexpected different modulation of the response to metformin after NB cell differentiation, which is not correlated to differentiation *per se* but directly depend on the expression level of NDM29 ncRNA.

We previously reported that NDM29 sensitizes SKNBE2 cells to several cytotoxic drugs (cisplatin, doxorubicin) by powerfully down-regulating MDR1 expression [21]. Thus, we verified whether the increased sensitivity of metformin observed in S1 cells might be related to NDM29-dependent alterations of the expression of molecules involved in the metformin interaction with the cells. In particular, we focused on the expression of the organic cation transporter 1 (OCT-1) that represents one of the main regulators of metformin cell internalization. However, in Western blot analysis we did not find significant differences in OCT-1 expression in pMock, S1 and S2 cells (data not shown), suggesting that different mechanisms should be responsible of the higher sensitivity to metformin of NDM29 expressing cells. Conversely, we observed that in SH-SY5Y OCT-1 expression was slightly reduced after metformin treatment (data not shown), possibly representing the molecular correlate for the lower sensitivity of these cells as compared to SKBNE2.

## Discussion

Besides being a first-line antidiabetic drug, metformin is currently under consideration for additional anticancer properties [33-35]. Recent reports evidenced TICs from different cancer types as the preferential targets of this drug [14-16]. These studies are in line with reports showing that also other antidiabetic drugs, such as PPAR-γ agonists exert cytostatic effects [36,37].

In this study we report, for the first time, an antiproliferative effect of metformin in a high-risk NB cell model. We show that metformin induces a significant reduction in the proliferation rate in two different NB cell lines (SKNBE2 and SH-SY5Y) characterized by different N-myc expression, although a higher sensitivity was observed in the first one. While we cannot provide a definitive answer for this difference, the observation that in SH-SY5Y cells, OCT-1 expression, which control the cellular internalization of this metformin, is down-regulated upon metformin treatment, could account for this difference.

In line with previous studies [15], this effect was related to a powerful inhibition of Akt activation, suggesting that the treatment with metformin could directly act on cell viability. In fact, beside an effect on cell cycle progression, as suggested by the reduction of DNA synthesis in [^3^H]-thymidine incorporation experiments, also increased cell death was observed in SYTOX blue staining experiments. Interestingly, metformin effects on cell survival did not were the results of activation of apoptosis since we did not detect nuclear shrinkage, caspase 3 activation or Bax up-regulation. Thus, from our data metformin elicits mainly a cytostatic effect that could indirectly cause cell death via apoptosis-independent pathways.

Interestingly, no changes in AMPK activity was observed in NB cells, although this kinase was reported to represent a key intracellular mechanism in metformin effects [38]. However, several studies suggested that mechanisms, others than AMPK activation, could mediate antiproliferative activity of metformin [39-41]. In particular, although several different mechanisms were identified in different tumoral cells, down-regulation of IGF-1 or other growth factor mediated autocrine signalling was suggested as main antiproliferative mechanisms regulated by metformin [42,43]. In turn, this effect could be responsible for the inhibition of pro-survival intracellular pathways, such as Akt, as we show in this study in NB cells and previously reported in GBM TICs [15]. However, further studies are still required to definitely address this issue.

Importantly, as shown in several other tumor cell types [14,15], metformin antitumoral activity was significantly higher in less differentiated NB cells, with a lower effect in differentiated cells (here we used ATRA-dependent differentiation).

Conversely, we found that metformin sensitivity is highly increased in NB cells in which differentiation is induced by NDM29 overexpression. This effect was strictly dependent on the expression levels of this ncRNA, which also correlate with the induction of NB cell differentiation. These data, and in particular the direct relationship between NDM29 expression and the reduction of NB cell viability induced by metformin, strongly suggest the possibility of a causal effect between the two events. At present we do not know the exact molecular mechanisms by which NDM29-dependent differentiation increases NB cell susceptibility to metformin, at odd with ATRA effects. In particular, since we previously reported that NDM29 overexpression inhibits MDR1 expression, representing a molecular mechanism that increases NB cell vulnerability to antimitotic cytotoxic drugs [21], we hypothesized that NDM29 expression might affect susceptibility to metformin inducing OCT-1 expression, thus increasing the amount of drug internalized into the cells and able to interfere with survival signals, such as Akt activity. However, here we report that SKBNE2 cells express similar OCT-1 content independently from the levels of NDM29 expression, and thus a different molecular mechanism needs to be identified.

## Conclusions

Our study for the first time demonstrates that metformin exerts antitumor activity against high risk NB cells, reducing cell proliferation and viability, via inhibition of Akt phosphorylation, showing higher sensitivity for less differentiated, highly proliferative cells. These data represent the starting point for further studies aimed to test the possible application of metformin in NB therapy. Moreover, as already shown for other cytotoxic drugs, the overexpression of NDM29, although inducing neuronal differentiation, powerfully sensitizes NB cells to metformin antiproliferative effects, suggesting that the pharmacological modulation of the expression of this ncRNA may represent a potential goal in the NB therapy. The molecular determinants by which the differentiation induced by NDM29, but not by retinoic acid, increases the antiproliferative activity of metformin will also represent a future goal in the translational research of novel NB therapies.

## Competing interests

The authors declare that they have no competing interests.

## Authors’ contributions

DC and AG made the vast majority of the experiments here reported and participated to the interpretation of results. RW performed Western blot analysis. TF contributed to the planning the experiments and the interpretation and discussion of the results and the draft of the manuscript. RC participated to the discussion of the results. AP planned the experiments, interpreted the results and wrote the manuscript. All authors read and approved the final manuscript.

## Authors’ information

Delfina Costa and Arianna Gigoni share first authorship.
